# Are women diagnosed with early pregnancy loss at risk for anxiety, depression, and perinatal grief?

**DOI:** 10.15537/smj.2022.43.9.20220291

**Published:** 2022-09

**Authors:** Levent Ozgen, Gulten Ozgen, Deniz Simsek, Burcu Dıncgez, Feyza Bayram, Ayten N. Mıdıkhan

**Affiliations:** *From the Department of Gynecological Oncology Surgery (Ozgen), Faculty of Medicine, Uludag University, and from the Department of Obstetrics and Gynecology (Ozgen, Simsek, Dıncgez, Bayram, Mıdıkhan), Bursa Yuksek Ihtisas Training and Research Hospital, University of Health Sciences, Bursa, Turkey.*

**Keywords:** abortion, anxiety, depression, perinatal grief

## Abstract

**Objectives:**

To examine the effects of early pregnancy loss on emotions such as depression, grief, or a sense of hopelessness, while investigating different types of diagnoses, hospital stays, and treatments.

**Methods:**

A prospective cohort epidemiological study was carried out in Bursa Yuksek Ihtisas Training and Research Hospital, Bursa, Turkey, between January and September 2019. The study included women diagnosed with early pregnancy loss classified into 3 groups: missed abortus, anembryonic pregnancy, and spontaneous abortion. The patients were screened via the Spielberger state-anxiety inventory (STAI-1) before initiating treatment. The Edinburgh postpartum depression scale (EPDS) and Perinatal Grief Scale (PGS) were also carried out in the first week of their hospital discharge.

**Results:**

The study was carried out with a total of 116 patients. The median gestational week of the patients was calculated at 9, their median hospital stay was 2 days, and their median dose of misoprostol was 800 mcg. The STAI-1 revealed that median values computed for women in all groups indicated moderate anxiety. The EDPS also demonstrated depression-positive median values for women in all 3 groups (EPDS>13). However, no statistically significant difference was noted in comparisons of the 3 groups apropos STAI-1, EPDS, and PGS.

**Conclusion:**

Moderate anxiety, depressed mood, and perinatal grief were found in women diagnosed with early pregnancy loss, regardless of the type of abortion.

The term early pregnancy loss or miscarriage denotes fetal death before the 14th week of gestation. Approximately 10% of pregnancies result in early pregnancy loss.^1,2^ Spontaneous abortion (SA) defines the loss of conceptus material before the 20th gestational week without any medical or surgical intervention.^3^ Fetoplacental material may be expelled without any complications but can also instigate extensive bleeding or endometrial infection in some cases.^3^ Spontaneous abortion is caused predominantly by chromosomal abnormalities associated with advanced age pregnancies.^4^

Missed abortion describes a non-viable fetus retained in the endometrial cavity.^4^ The diagnosis of such a miscarriage could be delayed and the condition may be detected during a later week of gestation, an event that could be more traumatic for patients. Missed abortion can be asymptomatic or patients can complain of vaginal bleeding or pelvic pain.

A blighted ovum, also called an anembryonic pregnancy, defines gestational sac development in the absence of an embryo.^4,5^

Early pregnancy loss is treated through the evacuation of the uterine cavity via surgical interventions such as aspiration curettage or via medical treatment generally utilizing misoprostol.^6^ Studies have amply compared these treatment modalities with respect to hospital stays, bleeding, or other complications. However, little is known regarding the psychological effect of these treatment models on the patients.

Women who experience early pregnancy loss suffer serious physical and psychological consequences such as grief, sadness, longing, social isolation, and guilt. Other family members may also be affected by psychological trauma.^7,8^ Initially, patients experience the trauma of pregnancy loss; subsequently, they endure the trauma of the treatment.

The present study intended to reveal the effects of different diagnoses of early pregnancy loss and discrete treatment modalities. It also evaluated the impact of the diagnosis-treatment interval on the psychiatric status of patients, for instance, their depression and anxiety symptoms.

## Methods

A prospective cross-sectional cohort study was carried out in Bursa Yuksek Ihtisas Training and Research Hospital, Bursa, Turkey, after obtaining approval from the local ethics committee (No.: 2011-KAEK-25 2020/01-11). The research was carried out based on the Helsinki principles. The study included patients diagnosed with early pregnancy loss and every participant tendered a written informed consent. The patients had been treated through medical or surgical interventions without any knowledge regarding the study. Physicians who had determined the medical or surgical treatment modalities were unaware of the study.

The World Health Organization (WHO) defines the term abortion as the expulsion of an embryo or fetus weighing 500 grams or less before 22 weeks, and the loss of the embryo is labeled as early pregnancy loss. Patients are categorized into 3 groups: missed abortus, SA, and anembryonic pregnancy. Women who had experienced recurrent pregnancy loss, hydatid moles, or extra-uterine pregnancies were excluded from this study as were patients with a history of depression, psychiatric illness diagnosis, and treatment.

The Spielberger state-anxiety inventory (STAI-1) scale was applied at the time of hospitalization to adjudge the anxiety status of every patient before initiating treatment. The Edinburgh postpartum depression scale (EPDS) and perinatal grief scale (PGS) measures of depression and grief, were obtained within the first week of a patient’s discharge from the hospital. Spielberger et al^9^ validated the STAI scale in 1970 as a means of quantifying the conditions of stress and anxiety. Subsequently, Öner et al^10^ validated the Turkish version in 1983. STAI-S and STAI-Tx separately evaluate the state and level of anxiety through 20 questions for each determination.^10^ The STAI cut-off established the overall scores of anxiety as none or low (20-37), moderate (38-44), and high (45-80).

The Edinburgh postpartum depression scale comprises 10 questions and is accepted worldwide. Each query is followed by 4 answer options scored between 0-3.^11^ It has been reported as more sensitive in detecting depression in patients when the cut-off score was calculated at 12-13.^12^ The present study applied the mentioned STAI and EPDS cut-off scores.

Toedter et al^13^ presented the PGS, which has been validated to determine grief after the perinatal loss. The Turkish version of the PGS contains 32 questions14 probing 3 subscales: i) active grief; ii) difficulty in coping; and iii) despair.^14^ The overall grief scores ranged between 33-165: higher total scores signify elevated levels of grief experienced by the patient.

### Statistical analysis

The Statistical Package for the Social Sciences, version 21.0 software for Windows (IBM Corp., Armonk, NY, USA) was used. Patients’ characteristics, demographics, and descriptive statistics were expressed as frequencies, median (minimum-maximum), mean ± standard deviation (SD) of the mean. Normality assumption was evaluated with the Shapiro-Wilk test. Kruskal-Wallis and ANOVA test were used for the analysis of continuous variables. Chi-square test was used for categorical data, and significance level was accepted as α=0.05.

## Results

The present study involved a total of 116 women who had experienced early pregnancy loss. The patients suffering missed abortus were 39.6%, SA were 39.6%, and anembryonic pregnancy were 20.7%.

The comparison of demographic variables and patient histories revealed no statistically significant differences in age, gravidity, parity, and the number of previous abortions. The median gestational week of the patients was calculated at 9 weeks and did not differ significantly (*p*=0.196) between the 3 established categories of subjects. Successful treatment entailed a median dose of 800 mcg of misoprostol in all groups, and this statistic also did not significantly differ across categories (*p*=0.841). The median hospital stay of participants of all groups was 2 days (Table 1).

**Table 1:** - Demographic characteristics of missed, spontaneous abortion, and anembryonic pregnancy groups.

Variables	Missed abortion (n=46)	Spontaneous abortion (n=46)	Anembryonic pregnancy (n=24)	*P*-values*
Age (year)	31.5 (20-44)	30 (20-44)	29.5 (19-42)	0.442
Gravida (n)	3 (1-10)	3 (1-10)	3 (1-10)	0.956
Parity (n)	1 (0-8)	1 (0-8)	1.5 (0-8)	0.936
Abortion (n)	1 (0-4)	1 (0-4)	1 (0-4)	0.960
Gestational week	9 (5-19)	9 (5-19)	9 (4-14)	0.196
Cytotec doz (qgr)	800 (0-3200)	800 (0-3200)	800 (0-2400)	0.841
Hospital stay (day)	2 (1-4)	2 (1-4)	2 (1-3)	0.196

Values are presented as median (minimum-maximum). *Kruskal-Wallis test

Anxiety, depression, and grief status were examined. The median score of STAI-S apropos missed abortion was 41.0 (26-54), 41.0 (26-54) for SA, and 41 (34-50) for anembryonic pregnancy. The 3 groups did not differ significantly in comparative examinations; however, the median scores of the women evinced moderate anxiety (*p*=0.964).

The median EPDS score was 16 (0-26) for all 3 groups. Patients registering a score above 13 in the EPDS are diagnosed as suffering from depression. The PGS median score for missed abortions was 100±33.63, 95.34±31.76 for SAs, and 93.12±35.24 for anembryonic pregnancy. The 3 groups did not differ significantly (*p*=0.623) in statistical comparisons, as demonstrated in Table 2.

**Table 2:** - Statistical differences between missed, spontaneous abortion, and anembryonic pregnancy groups in terms of Spielberger state-anxiety inventory (STAI-I), Edinburgh postpartum depression scale (EPDS), and perinatal grief scale (PGS).

Scales	Missed abortion (n=46)	Spontaneous abortion (n=46)	Anembryonic pregnancy (n=24)	*P*-values
STAI-1	41 (26-54)	41 (26-54)	41 (34-50)	0.964*
EPDS	16 (0-26)	16 (0-26)	16 (0-26)	0.829*
PGS, mean±SD	100±33.63	95.34±31.76	93.12±35.24	0.623^†^

Values are presented as median (minimum-maximum). *Pearson Chi-Square, †ANOVA

The STAI-S cut-off scores deemed anxiety in women as none or low (20-37), moderate (38-44), and high (45-80); no statistical difference was found between the anxiety levels exhibited by the 3 groups (*p*=0.573). The number of missed abortion patients diagnosed with depression via the EPDS was 67.4%, 73.9% for SA, and 83.3% for anembryonic pregnancy. Depression was detected in 85 (73%) patients who participated in the study; however, no statistical differences were found in comparisons of the scale of depression between the groups (*p*=0.357; Table 3).

**Table 3:** - Comparison between missed, spontaneous abortion, and anembryonic pregnancy groups degree of anxiety with STAI-1 scale and depression grades according to Edinburgh postpartum depression scale.

Groups	Anxiety level				Depression		
	None-less (20-37)	Moderate (38-44)	High (45-80)	*P*-value	(≤12 none)	(>13 present)	*P*-value
	n=22	n=60	n=34				
Missed abortion (n=46)	9 (19.6)	24 (52.2)	13 (28.3)	0.573*	15 (36.1)	31 (67.4)	0.357*
Spontaneous abortion (n=46)	7 (15.2)	27 (58.7)	12 (26.1)		12 (26.1)	34 (73.9)	
Anembryonic pregnancy (n=24)	6 (25.0)	9 (37.5)	9 (37.5)		4 (16.7)	20 (83.3)	

Values are presented as a number and precentage (%). *Pearson Chi-square test

## Discussion

The diagnosis of anxiety or depression may be deemed inessential for early pregnancy loss because patients do not feel fetal movements. It is also assumed that the connection between the fetus and the pregnant woman may not have developed. Nevertheless, the facts oppose such presumptions. The present study primarily intended to compare the diagnoses of different types of early pregnancy loss. However, all participating patients registered a median STAI-S score of 41, regardless of their classification. Thus, all patients presented with moderate anxiety without any statistical difference observed between the 3 groups.

Edinburgh postpartum depression scale was used to evaluate whether and to what degree the participating women were depressed. It was found that 85 of 116 (73%) patients registered scores above 16. The PGS also revealed that the median scores of women across all 3 groups evidenced moderate anxiety. These findings were crucial because early pregnancy loss is one of the most common pregnancy gravidity-related complications and can cause both physical and psychological symptoms.^15,16^ It is a traumatic and unexpected event that could form the initial trigger for disastrous psychological diseases. Socio-cultural factors affect the prevalence of depression in the general population.^17,18^ The present study ascertained the factors affecting levels of depression in women who experienced a miscarriage: the younger age, lower education level, advanced gestational week, marital status, number of previous miscarriages, and pregnancy obtained through assisted reproductive techniques were found to increase depression levels.^18^ Mitiso et al^18^ reported the influence of a subject’s education level was low. However, unlike their study, the participating patients in the present study were all married and living with husbands, and almost all their pregnancies had occurred spontaneously.

A contemporary study established a variance of between 30-50% in anxiety symptoms related to miscarriage and observed symptoms of depression in 10-55% of subjects.^17^ It reported that such symptoms appeared immediately after a miscarriage and lasted between 6 months and a year.^17^ Miscarriage often causes a period of intense emotional distress and also requires a grieving process. Untreated anxiety and depression negatively affect a person’s quality of life, human relations, and work-life, impair physical health, and increase suicidal tendencies.^19^ The present study revealed that early pregnancy loss was related to moderate anxiety and depression, regardless of the type of miscarriage. A guide published by the American College of Obstetricians and Gynecologists (ACOG) noted that maternal age and a history of previous pregnancy loss posed the highest risks for early pregnancy loss.^2^ According to this guideline, the rates were 9-17% between the ages of 20-30, and the incidence increased dramatically after the age of 35. The occurrence of miscarriages was 40% over the age of 40, and 80% over the age of 45.^2^ The median age of miscarriages in the missed and spontaneous abortion groups of the present study was 30 years and above, which aligned with the guideline. Other studies have reported that 15-20% of all pregnancies are lost due to spontaneous abortions, and missed abortions are common in the first trimester. This finding was also supported by the present investigation, in which the median gestational week of the participants at the time of miscarriage was 9 weeks.^20,21^ Farrell et al^23^’s valuable study declared that 11% of the women who experienced a miscarriage or an ectopic pregnancy suffered moderate to severe depression one month after treatment, the anxiety rate was 17% and depression rate was 5%, 9 months after the treatment. One study stated that a woman instinctively prepares her body and brain for motherhood once her pregnancy is diagnosed. Hence, women may experience intense feelings of emptiness, sadness, worries on re-pregnancy, and unworthiness when a pregnancy ends in miscarriage.^22^ No comparative statistical differences were found between the 3 groups in the present study; however, the PGS revealed that women in all groups registered median scores that established moderate anxiety. Additionally, the depression scale administered in the first week of hospital discharge revealed that 73% of the participating patients were depressed.

Volsgten et al^24^ evaluated the physical and mental health levels of Swedish women diagnosed with suffering spontaneous or missed abortions. To this end, they evaluated the post-abortion depression and perinatal grief of the women and their partners administering validation questionnaires one week after the treatment and 4 months afterward. Women were affected more deeply than their partners. Another study declared that emotional symptoms such as isolation stemming from the loss of a baby and other devastating events last even more than 4 months.^25^

### Study limitations

Our study has some limitations. Patients have been evaluated at the first week of treatment solely. The long-term effects have not been examined.

In conclusion, women diagnosed early pregnancy loss were at moderate risk of anxiety, depression, and perinatal grief, regardless of the type of diagnosis. These patients should not be underestimated with their current condition, and care should be taken to protect them from more intense diseases by following them closely.
